# Study of treatment outcome of patients of tuberculous cervical lymphedenopathy

**DOI:** 10.1186/1471-2334-14-S3-P42

**Published:** 2014-05-27

**Authors:** Nisha Mathew, Sunil Jadhav, Ashish Deshmukh, Hafiz Deshmukh, Shivprasad Kasat, Sandeep Dandin

**Affiliations:** 1 Mahatma Gandhi Mission's Medical College & Hospital, N-6 CIDCO, Aurangabad, Maharashtra, India

## Background

Tuberculosis lymphadenopathy is the most common form of extrapulmonary tuberculosis. The aim of the present study is to detect the duration of treatment required in patients with cervical lymphadenopathy.

## Methods

The patients attending the Department of Pulmonary Medicine OPD with cervical lymphadenopathy were screened. Detailed history, clinical examination, USG neck, FNAC, Mantoux test, biopsy, and chest X ray frontal view were performed and anti tuberculosis treatment initiated.

## Results

Total patients examined: 117, in the age group 10-70 years (mean age 40). Females affected – 89 (76.07%), Males - 28 (23.93%). Most common age group affected (M/F): 15-20 years: 37 (31.63%) patients. Biopsy was done in 73 patients (62.39%). Patients received Category-1ATT under RNTCP. After 6 months of therapy, 22 patients (18.81%) stopped treatment in view of non-palpable mass. Out of remaining 95 patients (81.19%), 14 patients’ (11.96%) lymph node size increased with slow regression, 17 patients’ (14.52%) liquefaction and repeated aspiration was done, 57 patients’ (48.72%) lymph node size showed regression less than 50%, 7 patients (5.98%) came up with new group of lymph nodes with size gradually increased. For all 95 patients, therapy was extended (continuation phase) for 2-3 months. 7 patients received ATT for 12 months.

## Conclusion

Extrapulmonary tuberculosis like tuberculous lymphadenopathy is benefited with extension of anti tuberculosis treatment.

